# Injectable platelet rich fibrin effect on laser depigmented gingiva: a clinical randomized controlled split mouth trial with histological assessment

**DOI:** 10.1590/1678-7757-2022-0307

**Published:** 2024-03-14

**Authors:** Suzan Seif Allah IBRAHIM, Ibrahim Abu MANDIL, Ola Mohamed EZZATT

**Affiliations:** 1 Ain-Shams University Faculty of Dentistry Department of Oral Medicine, Periodontology and Oral Diagnosis Cairo Egypt Ain-Shams University, Faculty of Dentistry, Department of Oral Medicine, Periodontology and Oral Diagnosis, Cairo, Egypt.; 2 Nahda University Faculty of Dental Medicine Department of Oral Medicine, Periodontology Oral Diagnosis and Radiology Beni Suef Egypt Nahda University, Faculty of Dental Medicine, Department of Oral Medicine, Periodontology Oral Diagnosis and Radiology, Beni Suef, Egypt.; 3 Ain-Shams University Faculty of Dentistry Central Lab of Stem Cells and Biomaterial Applied Research Cairo Egypt Ain-Shams University, Faculty of Dentistry, Central Lab of Stem Cells and Biomaterial Applied Research (CLSBAR), Cairo, Egypt.

**Keywords:** Hyperpigmentation, Diode laser, Submucosal, Mesotherapy, Platelet, Wound healing

## Abstract

**Objective:**

To determine whether intra-mucosal injection of injectable platelet-rich fibrin (i-PRF) can promote healing after Diode Laser Gingival Depigmentation (DLGD).

**Methodology:**

A total of 20 arch sites of hyperpigmented gingiva of 10 patients underwent DLGD. For each patient, two arch sites were randomly assigned for either intra-mucosal injection of i-PRF (G1-i-PRF) (n=10 sites) or no treatment (G2-Control): (n=10 sites). Wound Healing Score (WHS), patient satisfaction, and Pigmentation Index (DOPI) were measured at 1 week and 1 and 3 months postoperatively. Histological assessment of tissue specimens was performed at baseline and 1 week.

**Results:**

The percentage change in WHS at 1 week was significantly higher in G1 (58.34±15.43) compared to G2 (37.50±11.79). At day 1, 50% of patients in G1 were pain free compared with 75% in G2, who had mild pain. Mean DOPI decreased significantly at 3 months in both groups (P-value <0.001), without significant differences between groups. G1 specimens showed significantly higher epithelial thickness (P-value <0.001), as well as a higher number of blood vessels and less percentage of inflammatory cells.

**Conclusions:**

i-PRF demonstrated better clinical and histological healing potential and less patient discomfort compared to sites without treatment after DLGD. Registered at https://clinicaltrials.gov/ as (NCT05283668).

## Introduction

There are increasing dental esthetic demands for a pleasant appearance, with a healthy dentition and esthetically improved gingival component.^1^ The coral-pink gingival color is a reflection of the number and size of blood vessels, the width of epithelium, as well as the extent of keratinization.^2^ However, hyperpigmentation of the gingiva is a direct result of excessive melanin deposition within the basal and supra-basal oral epithelium.^3^

Gingival melanin hyperpigmentation is commonly physiologic. Nevertheless, etiological and pathological factors such as genetics, heavy metal poisoning, medications, endocrine disorders, malignant or benign lesions, smoking, and inflammation should not be excluded.^4^

Gingival depigmentation procedures imply the removal of the gingival hyperpigmented tissues together with a layer of the underlying connective tissue. The denuded connective tissue is subsequently allowed to recover by secondary intention.^5^ Occasionally, coverage of exposed connective tissue is suggested to minimize the post-operative hemorrhage and prevent surface trauma and external irritants on the wound surface, thus facilitating healing.^6^ Different depigmentation methods have been used, including the use of abrasion or scalpel/stripping surgical techniques, as well as advanced electrosurgery, radiosurgery, cryosurgery, and laser techniques.^7,8^

Numerous studies of gingival depigmentation techniques have been conducted to determine the most efficient and satisfying approaches for patients.^8,9^ Laser depigmentation is a relatively bloodless procedure since it coagulates and vaporizes tissues, also presenting wound tissue sterilization with minimal swelling and scarring.^10^ Moreover, diode laser showed better aesthetic results, less discomfort, and patient preference compared to other techniques.^11^ However, laser wounds are characterized by delayed healing with an average of three weeks possibly due to thermal tissue injury, which is usually followed by an increase in inflammatory response and subsequent delay in the initial organization of tissues.^12^

Different methods have been investigated to accelerate wound healing and improve patient satisfaction following gingival depigmentation procedure, including low-level laser treatments,^13^ ozonated oil,^14^ and bioactive materials, such as platelet concentrates.^15-17^

Injectable Platelet-Rich Fibrin (i-PRF) is produced at low speed centrifugation in the form of liquid for 10 to 15 minutes. Then, it is clotted in a gel with a high concentration of platelets, leukocytes, and growth factor concentration, which presents a slow and sustained release in the tissues.^18^ Thus, i-PRF may contribute to wound-healing processes by releasing variable growth factors, increasing vascularization, inducing the expression of collagen-1 mRNA, and transforming growth factor-β.^19^

Several studies have demonstrated that PRF in membrane form was a successful approach to protect the raw wound area and reduce healing time and patient discomfort when sutured over the gingival depigmented site.^15,16^ Moreover, other studies have documented the influence of i-PRF on increasing gingival thickness.^20,21^

To the best of our knowledge, no published clinical trial investigated the wound healing potential of i-PRF following laser gingival depigmentation as an easy-to-use platelet concentrate. Thus, this study was conducted to assess the clinical and histological effects of i-PRF on laser-depigmented gingival wound sites and its subsequent effect on patients’ satisfaction.

The research hypothesis was that injection of intramucosal i-PRF after laser gingival depigmentation would have a better healing score and patient satisfaction in terms of pain and esthetic perception compared with no treatment.

## Methodology

### Sample size calculation

The 2-sided statistical test of the null hypothesis was applied assuming that there is no difference between the effect of i-PRF injection after laser depigmentation on the wound healing score (WHS) [as a primary outcome], patient satisfaction, and Dummett-Gupta Oral Pigmentation Index (DOPI), as well as on epithelial thickness, number of blood vessels, and percentage of inflammatory cells [as secondary outcomes] compared to laser depigmentation alone. The patient’s arch site was assumed as the statistical comparison unit after merging the scoring values of the right and left quadrants. An alpha (α) level of 0.05, a Beta (β) level of 0.10 (power=90%), and an effect size (d) of (1.79) for WHS were set according to the results of Giannelli, Formigli, and Bani^22^(2014). The predicted sample size (n) was 14 samples, with 7 for each group. The sample size was calculated using G*Power software (version 3.1.9.7 from https://gpower.software.informer.com/3.1/). The sample size was increased to 20, with 10 in each group to compensate for dropouts.

### Eligibility criteria and patients’ recruitment

A total of 10 patients seeking cosmetic therapy for their gingival hyperpigmentation were recruited from the outpatient clinic of the Oral Medicine, Periodontology and Oral Diagnosis Department, Faculty of Dentistry, Ain Shams University from May 2022 to February 2023. Patients were screened to determine their eligibility according to the following criteria: Individuals considered Type 1 according to the American Society of Anesthesiology (ASA) status classification system, aged from 18 to 45 of both sexes,^23^ presenting diffuse physiologic melanin pigments in both upper and lower anterior gingiva with minimum Dummett-Gupta Oral Pigmentation Index (DOPI) of 3,^24^ and attached gingiva width of 1.5mm.

Exclusion criteria were individuals with active smoking habit, pregnant or nursing women, mental disabilities, or patients with active caries, periodontitis, or non-vital anterior teeth, and with a history of previous depigmentation operations were all excluded from the study.

### Study design

**Figure 1:**
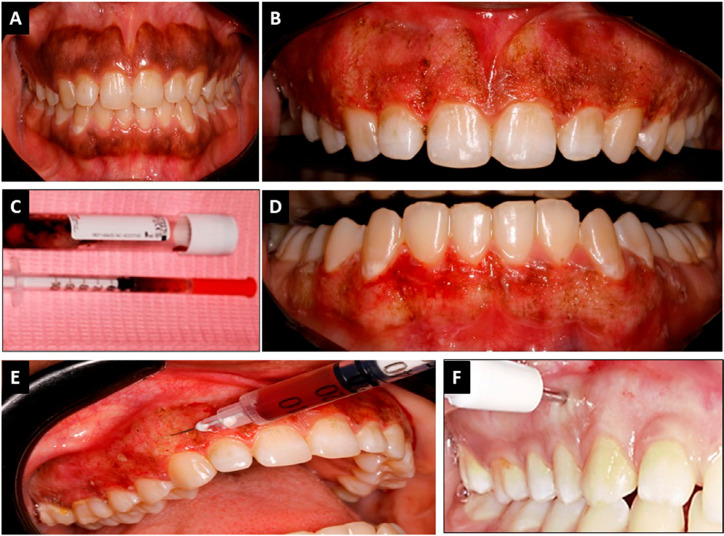
Clinical photographs depicting the study methodology; (A) Gingival hyperpigmented sites in the same patient at baseline (one maxillary and one mandibular). (B) Gingiva at maxillary arch immediately after depigmentation by diode laser, which was randomly assigned for Group 1 in this case; (C) i-PRF is produced in glass-coated plastic tubes and then loaded in a 29G needle. (D) Gingiva in mandibular arch immediately after depigmentation by diode laser which was randomly assigned for Group 2 (Control). (E) In Group 1, i-PRF was injected locally and at a depth of 1 mm parallel to the epithelium-connective tissue junction throughout the depigmented area of the maxillary gingiva. (F) One week after the surgery, a gingival sample was collected using a biopsy punch from the most distal point of each treated arch

The Consolidated Standards of Reporting Trials (CONSORT) specifications were applied in this two-parallel-arms, split-mouth, single-center, and assessor-blinded controlled randomized clinical trial. A total number of 20 arch sites of hyperpigmented gingiva were selected in patients who met the eligibility criteria; two in each patient, being a maxillary and a mandibular (Figure 1A). The two sites in each patient were then randomly assigned for two different treatment regimens using computer-generated random tables. The random allocation was performed by enclosing the group identification in sealed envelopes handled by a non-involved physician.

Due to the different intervention strategies used, it was impossible to blind the patient and the researcher. However, the researcher who performed the depigmentation procedure [I.A] was different from who provided the i-PRF injection [O.E], and the outcomes assessor was blinded to the type of assigned intervention [S.I].

### Treatment protocol

All eligible patients with pigmented gingiva in the upper and lower arch received oral hygiene instruction and professional scaling with ultrasound scalers in the preoperative phase.

The field block technique was used to anesthetize the pigmented gingival area in the upper and lower arch for each patient (Mepivacaine 2% in Levonordefrine 1:20000, Memphis Chemical and Pharmaceutical Industries, Cairo, Egypt).

A soft tissue diode surgical laser unit was adjusted as follows: 300 μm fiber tip diameter, wavelength of 940±10 nm, irradiation contact mode, and continuous wave with 2 W power (EpicX Diode Laser, Biolase, CA, USA). The laser tip was activated in contact and brushing motion was performed following the direction from the free gingival margin to the mucogingival junction and the interdental papilla was included. A gauze soaked with normal saline was used to remove epithelial remnants, hydrate the tissues, and improve visualization.^25^ The procedure was repeated over the pigmented area in the upper and lower arch until a thin layer of carbonized material appeared over a fresh non-bleeding surface (Figure 1B, D).

The i-PRF was produced by a collection of 9 mL of venous blood in plain plastic tubes (VACUETTE^®^, Greiner Bio-One White Top Tubes 9 ml, 16x100 white cap-black ring, non-ridged) then immediately centrifuged at 700 rpm, 60.4 relative centrifugation force (RCF), 71 g-force at the clot (RCF-clot), 109.8 g (RCF-max), 48.2 g (RCF-min) for 3 mins at room temperature using the certified standardized centrifuge machine; Intra-spin, 41.3° rotor angulation, 110 mm radius (Duo Quattro Centrifuge, Process for PRF, Nice, France).^26-28^ The 1 mL upper plasma layer was then collected in a 29 Gauge, 5/16’’, 1cc, 0.33×8 mm needle to be injected (Figure 1C).

The laser-depigmented arches in each patient were randomly assigned into two groups;

Group 1 (G1***-***i-PRF), in which the prepared and loaded i-PRF was regionally inserted, beveled upward, and injected at 1 mm depth parallel to the epithelium-connective tissue junction across the depigmented area (2-3 mm apart points, 0.1 ml for each) (Figure 1E).^29^

Group 2 (G2*-*Control), in which the laser depigmented sites were left to heal without using periodontal dressings. All intervention procedures were performed by the same operator**.**

To prevent mechanical damage and promote re-epithelialization, patients were advised not to brush their teeth on the day of surgery and not to eat hot or spicy food for the first 24 hours. On day 2, regular toothbrushing and interproximal cleansing were encouraged.^30^

### Assessment

Clinical assessment was performed by the same investigator; Wound Healing Score (WHS) was measured at baseline and after one week considering: 1) Total re-epithelialization, 2) Partial re-epithelialization, 3) Ulcer, and 4) Tissue defect/necrosis.^22^

Gingival pigmentation was measured at baseline, 1 week, 1 month, and 3 months using Dummett-Gupta Oral Pigmentation Index (DOPI) considering: 0) pink tissue without clinical pigmentation, 1) mild light brown tissue pigmentation, 2) medium brown or mixed brown and pink tissue pigmentation, and 3) Deep brown/blue-black tissue pigmentation.^24^ Additionally, the degree of pain associated with the study intervention and the patient’s satisfaction with the procedure aesthetic outcomes were measured using a satisfaction questionnaire.^31^

At one week post-operatively, histological assessment of gingival healing was performed by collecting two tissue samples from each patient (2 mm wide and 1 mm deep). These samples were collected by the same operatorusing a biopsy punch (Kai Industries Co., Japan) from the most distal site of the depigmented area unilateral at each arch site—one from the upper arch and one from the lower arch for each patient— being careful not to expose the periosteum or approximate the gingival margin (Figure 1F).

Biopsied tissues were dried in graded ethanol before being embedded in paraffin. The 10% (weight/volume) formaldehyde solution in 0.2 ml phosphate-buffered saline (pH 7.4) was used to fix the tissues. Deparaffinized and rehydrated slices that were 4-5 μm thick were produced before histological staining with hematoxylin and eosin. Using a digital camera (C5060, Olympus, Japan) mounted on a light microscope (BX60, Olympus, Japan), photomicrographs of three microscopic fields were taken for each section. Using the Image J software (Version 1.41a National Institutes of Health, Bethesda, MD), digital images at original magnifications of 20X and 40X were then analyzed for mean epithelial thickness, number of inflammatory cells, and percentage area fraction of blood vessels.^16^ All histological procedures and analyses were performed by an expert specialist blinded to the type of intervention in Oral Pathology Laboratory, Faculty of Dentistry, Ain Shams University, Cairo, Egypt. Analysis of the following morphologic parameters using the tools and plug-ins of the software was performed according to Vasanthi, et al.^32^ (2022) as follows:

Mean epithelial thickness was measured in micrometer (μm) from the most superficial point of the epithelium to the junction between epithelium and connective tissue at three different points by selecting Analyse > Measure tool and the mean was calculated.

The number of inflammatory cells per field and mean of the three fields were obtained manually with the help of a [cell counter > plug-in > Image J]. The severity of inflammation was evaluated among different groups based on the following criteria:

Mild: less than 100 inflammatory cells per field

Moderate: 100 to 250 inflammatory cells per field

Severe: more than 250 inflammatory cells per field.

The blood vessels were traced with the free hand tool to measure their area and the mean values were calculated as mean vascular. The mean percentage area fraction of blood vessels was estimated from the total area and blood vessel area in percentage.

### Compliance with ethical standards

The study received the approval from the Research Ethics Committee at the Faculty of Dentistry, Ain Shams University (FDASU-REC D076302), and was conducted in compliance with the Declaration of Helsinki (2013), registered at www.clinicaltrials.gov (NCT05283668), applying the Consolidated Standards of Reporting Trials (CONSORT) specifications. All patients were given full details of the study procedures and signed an informed consent form to undergo treatment and biopsy procedures.

### Statistical analysis

Kolmogorov-Smirnov and Shapiro-Wilk tests of normality were performed to explore numerical data by checking the distribution of data. The parametric data were presented as mean and standard deviation (SD) values. Qualitative data were presented as frequencies and percentages. To compare the mean values between groups, the independent-sample t-test was used. To study the changes by time in outcomes within each group, the paired-sample t-test was used. Fisher’s exact test was used to compare groups in frequencies and percentages (%) of responses to satisfaction questionnaire questions. The significance level was set at 0.05. IBM SPSS Statistics for Windows (Version 23.0. Armonk, NY: IBM Corp) was used for statistical analysis

## Results

**Figure 2:**
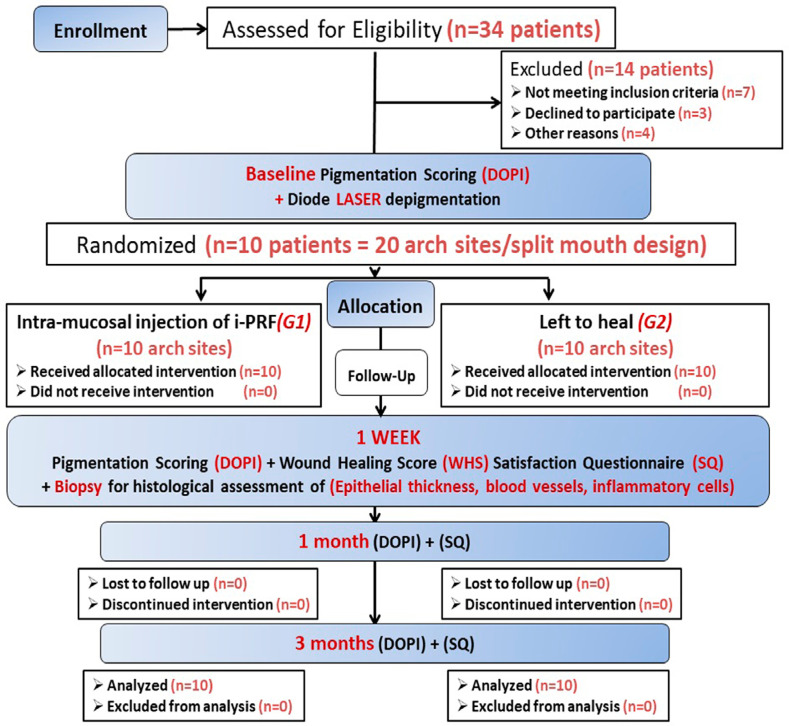
A CONSORT flow chart for the study showing how participants were enrolled and when outcomes are evaluated

**Figure 3:**
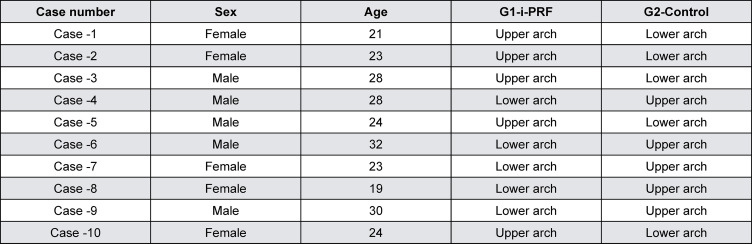
The sociodemographic characteristics of included participants

According to the CONSORT flow chart (Figure 2), all participants followed the treatment protocol without experiencing any major side effects or dropouts during the research period. The mean age of participants was 25.2±4.17 years with an age range from 19 to 32 years and included five males (50%) and five females (50%). Figure 3 presents sociodemographic characteristics of included participants.

### Wound healing score (WHS)

Immediately after interventions, at day 1, the mean WHS was 3.00±0.00, indicating a complete loss of epithelium (ulceration) in both groups. However, WHS decreased significantly after one week in both G1 and G2, being 1.25±0.46 (p=0.01) and 1.88±0.35 (p=0.008), respectively, indicating that most cases in both groups showed total re-epithelization. Moreover, when comparing the mean percentage change of WHS after one week between the two groups, we found that G1 mean percentage reduction was 58.34±15.43, which was significantly higher than that of G2, being 37.50±11.79 (p=0.049).

### Patient satisfaction score

On the day of the depigmentation procedure following the complete resolution of the local anesthetic effect, 50% of G1 patients were reported to be pain-free, whereas 70% of G2 reported having mild pain. All patients experienced no pain and noticed a marked cosmetic change during the first week after treatment but the differences between groups were not significant regarding frequencies of responses to all questions, as shown in Table 1.

**Table 1:** Comparison between groups in responses to satisfaction questionnaire questions

Questionnaire Questions	G1-i-PRF	G2-Control	P-value
	**(n = 10)**	**(n = 10)**	
**Q1: Do you think the treatment was painful?**			
Mild	6 (60.0%)	5 (50.0%)	
Moderate	4 (40.0%)	4 (40.0%)	0.484^ns^
Severe	0 (0%)	1 (10.0%)	
**Q2: Did you feel pain on the day of the treatment?**			
Not at all	5 (50.0%)	2 (20.0%)	
Mild	5 (50.0%)	7 (70.0%)	0.129^ns^
Severe	0 (0%)	1 (10.0%)	
**Q3: Have you felt pain during the 1st week following the treatment?**			
No	10 (100%)	10 (100%)	
Mild	0 (0%)	0 (0%)	1^ns^
Severe	0 (0%)	0 (0%)	
**Q4: Have you noticed a cosmetic change in the 1st week of treatment?**			
No	0 (0%)	0 (0%)	
Moderate	0 (0%)	0 (0%)	1^ns^
Marked	10 (100%)	10 (100%)	
**Q5: Did you notice a cosmetic change after 3 months of treatment?**			
No	0 (0%)	0 (0%)	
Moderate	2 (20.0%)	0 (0%)	1^ns^
Marked	8 (80.0%)	10 (100%)	
Q**6: Do you think the treatment met your expectations?**			
No	0 (0%)	0 (0%)	
Yes	10 (100%)	10 (100%)	1^ns^
Over and above	0 (0%)	0 (0%)	
**Q7: Would you repeat the treatment if needed?**			
No	2 (20.0%)	2 (20.0%)	
Yes	8 (80.0%)	8 (80.0%)	1^ns^
Over and above	0 (0%)	0 (0%)	

ns = non-significant (p>0.0) according to Fisher's exact test.

### Dummet oral pigmentationindex (DOPI)

**Figure 4:**
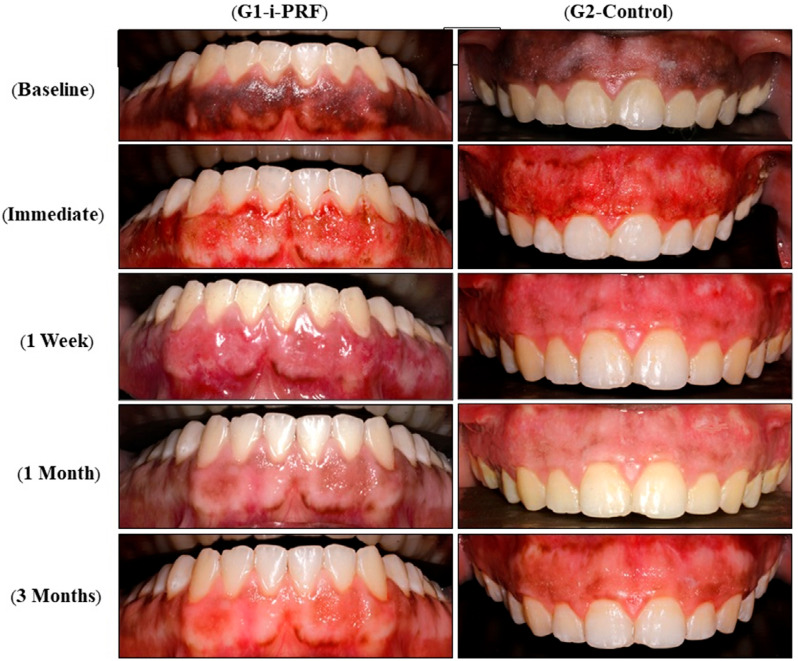
Intraoral photographs showing clinical changes of gingival sites in Group 1 and Group 2 at baseline and immediately after laser depigmentation, then at 1 week and 1 and 3 months

Gingival tissues with spreading of pinkish areas were observed in both groups at 1 week and 1 and 3 months postoperatively. However, the vascularized red thick gingival tissue was markedly observed within the first week in the i-PRF group (Figure 4). Occasionally, mild light brown localized remnants of pigmented tissues were left at periphery of arch sites over bony prominences or at thin gingival margins and mostly located underneath the smile line and, thus, the DOPI scores for these arches was not affected. The mean DOPI decreased significantly (p<0.001) from baseline (2.75±0.46) to 1 week after treatment (0.00±0.00) and this score was maintained in both groups until 1 month. However, the mean DOPI increased at 3 months in G1 (0.62±0.52) and in G2 (0.75±0.46), with no significant difference between values recorded at 3 months and values recorded at different follow-up intervals (p>0.05). G1 showed a higher mean percentage reduction in pigmentation score within 3 months (79.17±17.25) compared with G2 (72.92±17.68) but the difference was not significant (p=0.504).

### Histological analysis

**Figure 5:**
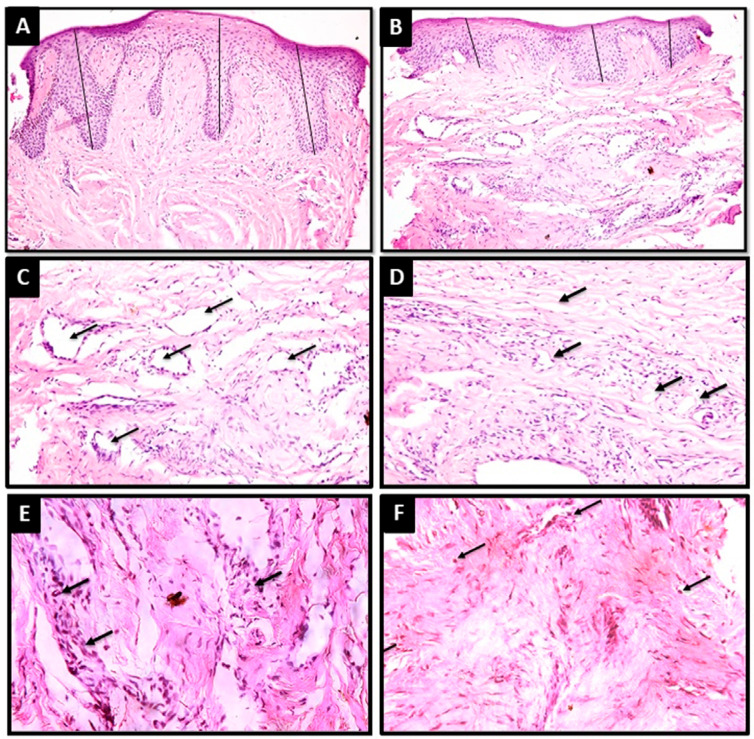
Photomicrographs of tissue specimens stained with hematoxylin and eosin after 1 week showed higher epithelial thickness (black lines) in Group 1 (A) than in Group 2 (B) (H&E, Original magnification 20x). The number and size of blood vessels (black arrows) were also higher in Group 1 (C) than in Group 2 (D) (H&E, Original magnification 40x). Photomicrographs also showed infiltrating inflammatory cells (black arrows) in both Group 1 (E) and Group 2 (F) (H&E, Original magnification 40x)

Epithelial thickness in G1 presented a significantly higher mean value in micrometers (853.28±126.70 μm) compared with G2 (460.14±71.02 μm) (p<0.001), as observed in Figure 5. The mean area fraction of blood vessels in G1 (24.62±2.07) was higher than that of G2 (22.38±4.07) but not considered significant (p=0.234), as shown in Figure 5. After 1 week of treatment, the G1 group presented two cases (20.0%) of severe inflammatory cell infiltrate and eight cases (80.0%) of mild infiltrate. In G2, three cases (30%) showed severe infiltrate, five cases (50%) showed moderate infiltrate, and two cases (20%) showed mild infiltrate. Figure 5 presents the inflammatory cells in photomicrographs from both groups.

## Discussion

In this study, to the best of our knowledge, intra-mucosal injection of i-PRF was assessed for the first time as an autologous bioactive wound healing inducer following laser gingival depigmentation in terms of clinical efficacy, histological changes, as well as patient satisfaction. Moreover, out of different previously tested platelet concentrates, i-PRF was chosen since it can be used in a liquid state before the formation of a fibrin clot (coagulation) without the use of anti-coagulants, making it entirely natural and easy to be injected. Every effort was taken to standardize the i-PRF protocol with the specification of all centrifugation parameters as recommended recently in literature.^27^

Miron, et al.^33^ (2020) evaluated 24 protocols for the production of platelet-rich fibrin and recommended the use of a horizontal centrifuge with lower speed and time for improved separation of blood cell and higher concentration of growth factors. However, the protocol to produce i-PRF used in the current study was chosen for being the most commonly used protocol in clinical trials.

The wound healing score used in our study was reported to be easy to use for researchers, safe for patients, suitable to various wound types, and tested to be reliable and precise without the need for special or expensive equipment.^34^ To evaluate the pain and cosmetic changes, a patient satisfaction questionnaire was used to fulfill all the criteria of evaluation, including pain experienced during and post-treatment, as well as cosmetic changes during the follow-up period and how the treatment met their expectations. Furthermore, histological analysis for epithelial thickness, number of blood vessels, and percentage of inflammatory cells in tissue specimens were performed at 1 week to detect early changes in the healing process as demonstrated by experimental models.^12^

The selection of the biopsy site to be the most distal area in the arch was justified by avoiding the anterior esthetic zone with a relatively thinner total gingival thickness to avoid the possible effect of biopsy on cosmetic and/or pain perception and to avoid the risk of bone exposure. However, on a microscopic anatomy level, the gingival epithelial thickness was not variable regarding oral epithelium in the premolar-molar area.

The study demonstrated that the mean percentage reduction of wound healing score was highly significant in G1*-i-PRF* compared to G2*-Control*, supporting our initial hypothesis. These results are comparable to those documented by Debnath and Chatterjee^16^ (2018) who compared PRF membrane to periodontal dressing on the third and fifth days following abrasion depigmentation technique. Our outcomes are also comparable with Dahiya, et al.^15^ (2019) and Bansal, et al.^17^ (2016) who used scalpel technique.

The previous clinical results of i-PRF wound healing potential have been supported by histological findings as G1 specimens showed significantly higher epithelial thickness, a higher mean area fraction of blood vessels, and a higher percentage of cases with mild inflammatory cell infiltrates compared to spontaneous healing in G2 specimens. Other studies have demonstrated these same findings using PRF membranes.^15-17^

The observed healing potential of i-PRF in the current study both clinically and histologically can be attributed to its fibrin gel, which acts as a matrix that supports platelets and leukocytes and carries the main angiogenesis soluble factors, such as basic fibroblast growth factor, angiopoietin, platelet-derived growth factor, and vascular endothelial growth factors. Fibrin matrix also guides the coverage of injured tissues by affecting the metabolism of epithelial cells and fibroblasts so that epithelial cells around the wound’s margins produce basal and lateral extensions towards the wound side. Additionally, the fibrin matrix allows for the rebuilding of fibrin in connective tissue and acts as a net for stem cells. To stimulate healing, i-PRF provides angiogenesis, epithelial cover, and immunity at the site of the lesion.^19,20,35^

Patients in this split-mouth trial design were asked to score their pain and cosmetic perception for each arch individually. The only observed difference between groups was on the day of the depigmentation procedure following the complete resolution of the local anesthetic effect, in which 50% of i-PRF-treated patients were pain-free compared with 70% in the control group who had mild pain. It was also noticed that patients reported no difference between the two techniques regarding treatment expectations and were willing to repeat the treatment if needed, indicating that i-PRF injection following laser depigmentation was simple and achieved patient compliance. However, these results differ from other studies that applied PRF membranes following surgical depigmentation techniques, as they documented that pain scores were lower with the use of platelet concentrates.^15-17^ Early epithelization and anti-inflammatory effect of i-PRF probably caused covering and less stimulation of peripheral nerve endings in laser-exposed connective tissue.

The higher mean percentage reduction in pigmentation score in the i-PRF group compared to the control group was not significantly different. However, it could be indirectly due to increased epithelization and vascularization. Furthermore, the direct effect could be attributed to the presence of transforming growth factor (TGF)-β1, 2 and epidermal growth factor (EGF) in i-PRF, which were found to decrease melanogenesis. TGF-β1 delays extracellular signal-regulated kinase activation in a concentration-dependent manner,^36^ whereas EGF inhibits prostaglandin-E2 (PGE2) expression and tyrosinase enzyme activity in melanocytes.^37^ Similar results have been documented with the use of intradermal platelet concentrates for facial hyperpigmentation alone or in combination with laser therapy,^38^ suggesting possible future research in using i-PRF as a natural depigmenting agent for gingiva and comparing it with other pharmacological agents as ascorbic acid.

The study was limited by the short follow-up period and relatively small sample size, as well as the inability to implement double-blinding for the intervention. Therefore, further clinical trials to detect possible long-term effects of repeated injections of i-PRF, as well as to compare it with other platelet concentrates or topical wound healing promotors on esthetic outcomes are recommended.

## Conclusions

Based on the results of the current study and considering its limitations; the following conclusions were drawn:

Local injection of i-PRF as a biological mediator following laser depigmentation enhanced the normal healing process, which was confirmed by histological analysis in the first week. It also reduced patient discomfort and pain feeling, thus presenting a positive effect on patient satisfaction with this technique.

Immediate intramucosal injection of i-PRF as an adjunct to laser depigmentation did not significantly influence the pigmentation score in the first three months.
